# Inter-correlation of risk factors among heart patients

**DOI:** 10.3934/publichealth.2020030

**Published:** 2020-06-10

**Authors:** Md Jahoor Alam, Abdullah Ibrahim Alnafeesah, Mohd Saeed

**Affiliations:** 1 Department of Biology, College of Science, University of Ha'il, Ha'il, KSA; 2 Department of Clinical Laboratory Sciences, College of Applied Medical Sciences, University of Ha'il, Ha'il, KSA

**Keywords:** risk factors, heart disease, inter-correlation, diabetes, statistical analysis

## Abstract

Cardiovascular disease is a well-known and widely studied disease. Epidemiological studies indicated that the mortality rate due to cardiovascular diseases increases every year. According to the WHO (world health organization) report, cardiovascular disease is one of the most prevalent non-communicable diseases in Saudi Arabia. Moreover, the Ministry of Health, Kingdom of Saudi Arabia, also reported that 42% of the non-communicable disease death is associated with cardiovascular disease in Saudi Arabia. Various reports suggest that heart disease is associated with several risk factors. Moreover, diabetes is one of the high-risk factors. Clinical result suggests a good association between diabetes and cardiovascular complications. In the present work, we focus on some of the important risk factors responsible for heart disease such as weight, blood pressure, age, and diabetes. A set questionnaire, which includes all the parameters associated with cardiovascular disease, was prepared. Data collected from the heart patient's records of different hospitals in Al-Qassim, Saudi Arabia. We applied statistical tools to analyze the data. Our results shows very interactive and significant pattern. We found significant inter-correlation between the different risk factors. In conclusion, the inter-correlation among different risk factors of heart disease is found. It is suggested that both pre and post-heart patients should be more concern about these risk factors. Moreover, this study can be used for further research, and, will increase people awareness regarding their healthy choice.

## 1. Introduction

Heart disease is well known as heart and blood vessel disease. Heart disease is responsible for a large number of deaths worldwide. It reduces the quality of life and also costs to the health system and economy [1]. The prevalence of heart disease is increasing worldwide [2].

Various reports indicate that heart disease can be prevented or treated with healthy lifestyle choices [3]. Experimental and theoretical works reported that mortality due to heart diseases increases every year globally. Heart disease found to be very serious non-communicable disease in the United States and most European countries and reported as a leading cause of death [4]. The latest WHO report suggests that the majority of killer diseases in the Kingdom of Saudi Arabia (KSA) are non-communicable, chronic diseases. Moreover, the ministry of health, Kingdom of Saudi Arabia, announced that coronary heart disease constitutes one of the main health problems in Saudi Arabia. According to the ministry report, heart disease represents the third most common cause of hospital-based mortality second to accident and aging. The prevalence of diabetes in the Kingdom is at an alarming level [5]. Over 25 percent of the adult population is suffering from diabetes, and that figure is expected to more than double by 2030 [6]. According to the World Health Organization (WHO), Saudi Arabia has the second-highest rate of diabetes in the Middle East and is the seventh highest in the world [7]. It is reported that heart disease is associated with several risk factors. One of the most reported risk factor is diabetes. A recent clinical result suggests that a high degree of association between diabetes and cardiovascular complications [8]–[11]. In the present work, we focus on some of the important risk factors such as weight, blood pressure, age, and diabetes, which are reported to be responsible for heart disease. However, a few works are published on clinical data for the interrelation between heart disease and risk factor, still there not much report published regarding intercorrelation among risk factors of heart disease. In this paper, we presented the intercorrelation among the risk factors using statistical measurement in clinically data collected from Al-Qassim region, Kingdom of Saudi Arabia.

## 2. Material and methods

This is a prospective cross-sectional study, which is a suitable method for calculation of the prevalence of conditions. First of all, we prepared a set of survey questionnaire which includes all the parameter associated with heart disease. Data of the 80 patients were collected from heart patients record of different hospitals in AlQassim (Central region), Kingdom of Saudi Arabia. Moreover, from several parameters, we excluded other parameters except for diabetes, blood pressure, weight, age, and gender. Patients were divided into different age groups (<20, 21–30, 31–40, 41–50, 51–60, 61–70 and 71–90). The age and sex of those affected with heart disease were noted. We calculated the percentage of different age groups in the total population and also the percentage of the affected population in different age groups in the total population. A suitable questionnaire was prepared with the aim to include all the inclusion criteria related to important risk factors of heart disease.

Statistical technique: The results are presented in frequencies and percentages. The Pearson correlation and t-test statistical measures are used for the association between risk factors. The p-value < 0.05 was considered significant. All the analysis was carried out on the SPSS 16.0 version (Chicago, Inc., USA).

## 3. Results and discussions

**Figure 1.**
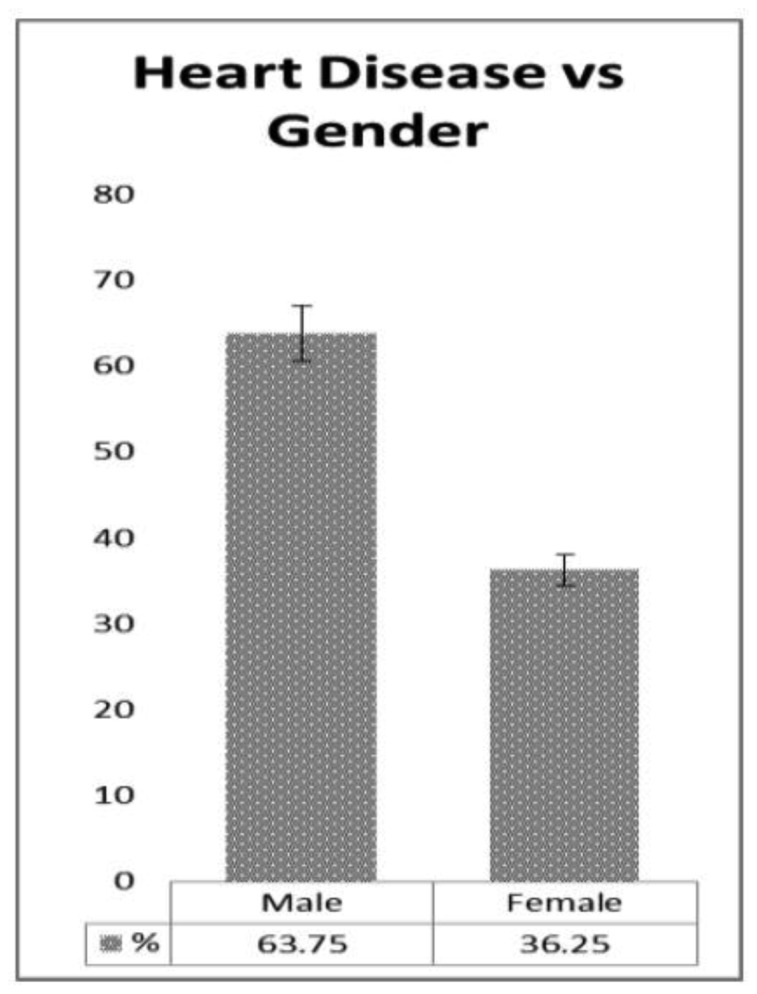
Heart disease patients according to gender.

**Figure 2.**
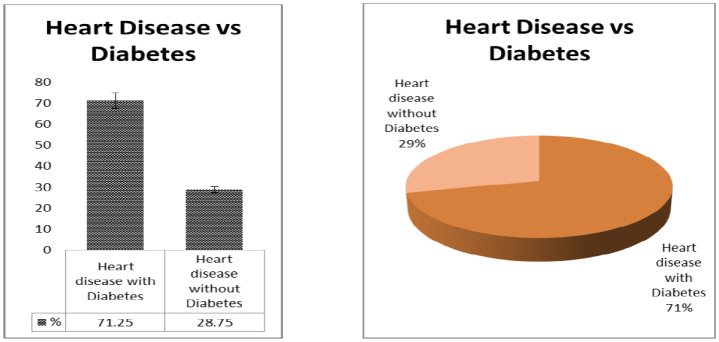
Heart disease patients with diabetes and without diabetes.

**Figure 3.**
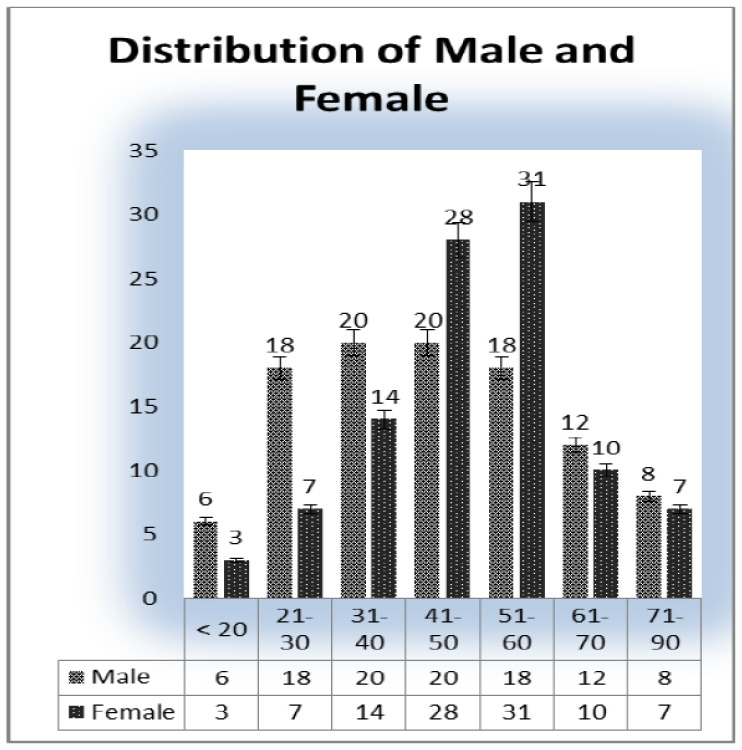
Heart disease patients according to with respect to age and gender.

**Figure 4.**
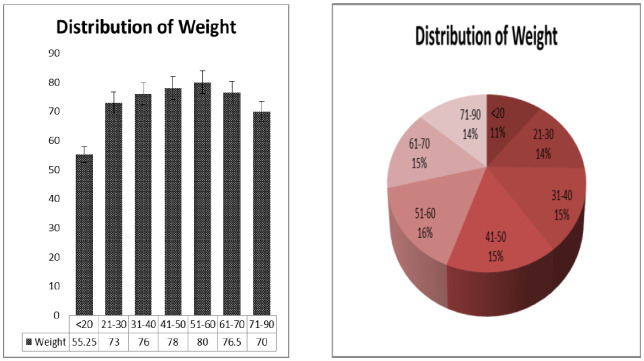
Over all distribution of weight of the heart patients with respect to different age group.

**Figure 5.**
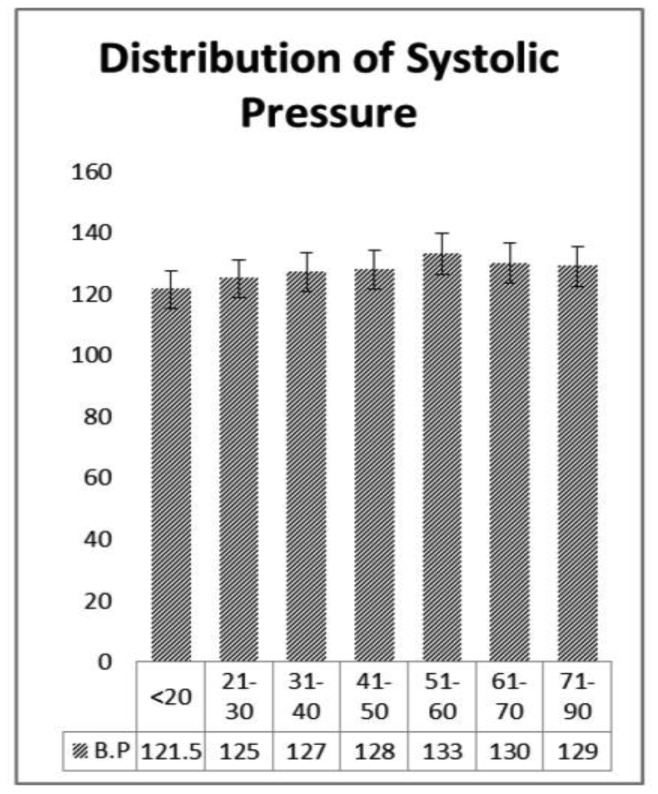
Plot for heart disease patients with diabetes and without diabetes.

**Figure 6.**
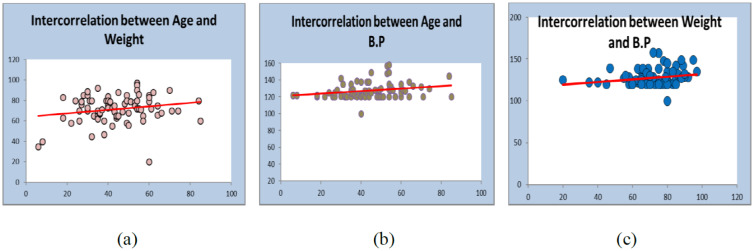
Plot for intercorrelation between different risk factors. (a) It indicates intercorrelation between age and weight; (b) It indicates intercorrelation between age and systolic blood pressure; (c) It indicates intercorrelation between weight and systolic blood pressure.

**Figure 7.**
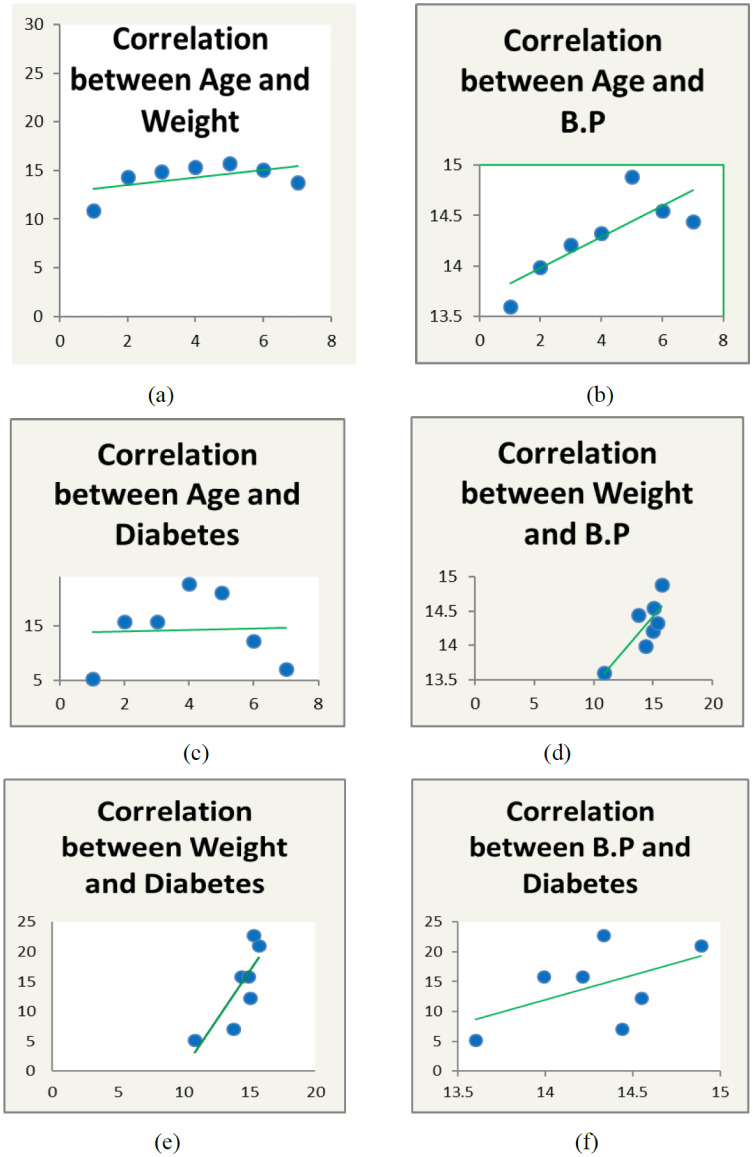
Inter-correlation among heart disease patients for different risk factor (Average). (a) Indicates intercorrelation between age and weight; (b) Indicates intercorrelation between age and systolic blood pressure; (c) Indicates intercorrelation between age and diabetes; (d) Indicates intercorrelation between weight and systolic blood pressure; (e) Indicates intercorrelation between weight and diabetes pressure; (f) Indicates intercorrelation between systolic blood pressure diabetes.

Weight and blood pressure of the patients is widely responsible for heart disease [12]. We presented a distribution table for risk factors of heart disease such as age, weight, and blood pressure with mean and standard deviation (as shown in Table 1). The average age is found to be 44.48 years with deviation of 15.64 years. Similarly, the average weight of the sample is 71.76 kg with deviation 13.45 kg, and the average blood pressure of the patients are 127.57 mm/Hg with deviation 9.57 mm/Hg. Further, in Table 2, the distribution of heart patients in male verses female is shown. It is found that more than half of the patients were males (63.75%) and lesser number of females (36.25%) as shown in Figure 1. In Table 3, distribution of heart patients with and without heart disease is shown. It is founds that heart patients with diabetes is higher (71.25%) and without heart disease is lesser (28.75%), as shown in Figure 2. This indicates diabetic patients are in high risk of heart disease. A cross-sectional analysis of heart patients' age group (<20, 21–30, 31–40, 41–50, 51–60, 61–70 and 71–90) verses weight, systolic pressure and percentage of diabetic patients are shown in Table 4. In Figure 3, the distribution of heart patient in male verses female with respect to different age groups is shown. It is noticed that in age group 41–50 and 51–60, females are more prone than males. We presented the distribution of weight among the heart patients according to their age group as shown in Table 4 (in Figure 4). It is observed that the weight of heart patients is normally distributed. It is noticed at the age group of 51–60 weight is comparatively higher than age groups. Similarly, we observed that systolic blood pressure of the age group 51–60 is very high, as shown in Figure 5. Further, we observed percentages of diabetes cases across the different age groups. We noticed that in the age group range 41–50, the percentage of diabetic cases is more than the other age groups [13] as shown in Table 4. Further, we plotted inter-correlation among the risk factors of heart disease. In Figure 6, intercorrelation has been shown in-between age and weight, between age and systolic blood pressure, between weight and systolic blood pressure. It is noticed that correlation are positive and significant at p < 0.05 (as shown in Table 5). Similarly, we plotted (as shown in Figure 7) inter-correlation among heart disease patients for different risk factors with respect to different age groups, average weight, average blood pressure, and percentage of diabetic patients, as shown in Table 6. Moreover, in Figure 7, a positive correlation has been observed in-between age and weight, between age and systolic blood pressure, between age and diabetes, between weight and systolic blood pressure, weight and diabetes, systolic blood pressure and diabetes. It is noticed that the correlation between age and blood pressure is high and statistically significant at p < 0.05. Similarly, the correlation between weight and blood pressure is very high (r = 0.816) and statistically significant at p < 0.05. Further, It is also noticed that the correlation between weight and diabetes is very high (r = 0.815) and statistically significant at p < 0.05.

**Table 1.** Distribution of risk factors of heart disease.

Risk Factors	Mean ± Std
Age	44.48 ± 15.64
Weight	71.76 ± 13.45
Blood Pressure	127.57 ± 9.57

**Table 2.** Distribution of heart patients' gender wise.

Gender	Number	%
Male	51	63.75
Female	29	36.25

**Table 3.** Distribution of heart patients with and without heart disease.

Cases	Number	%
Heart Patients with Diabetes	57	71.25
Heart Patients without Diabetes	23	28.75

**Table 4.** Distribution of weight, blood pressure and diabetes with respect to different age group.

Age (Years)	Weight (kg)	Systolic B.P (mm/Hg)	Diabetes (%)
<20	55.25	121.5	5.26
21–30	73	125	15.79
31–40	76	127	15.79
41–50	78	128	22.81
51–60	80	133	21.05
61–70	76.5	130	12.28
71–90	70	129	7.02

**Table 5.** Correlation 1.

Attributes	Pearson-correlation
Age v.s. Weight	0.198 *
Age v.s. Blood Pressure	0.238 *
Weight v.s. Blood Pressure	0.220 *

Note: * p-value < 0.05 (Significant).

**Table 6.** Correlation 2.

Attributes	Pearson-correlation
Age** v.s. Weight**	0.276
Age** v.s. Blood Pressure**	0.645*
Age** v.s. Diabetes**	0.186
Weight** v.s. Blood Pressure**	0.816*
Weight** v.s. Diabetes**	0.815*
Blood Pressure** v.s. Diabetes**	0.516

Note: * p-value < 0.05 (Significant); ** Average.

## 4. Conclusion

According to several published reports, the number of heart disease patients increases every year in Saudi Arabia [14]. The present work is focused on found out inter-correlation among different risk factor of heart disease. It is concluded from our results that risk factors which are associated with heart disease, can be a marker of the disease. The distribution of these factors shows a normal distribution. Moreover, Diabetes is found as one of the important risk factors of heart disease. Diabetes is prevalent in AlQassim region, and this increase with an alarming rate needs an urgent program aiming for people to learn about risks and warning signs of diabetes, to take actions to prevent the disease and seek healthcare in case they develop diabetes and to encourage in other aspects of getting a healthy lifestyle. Diabetes risk measurement must be incorporated into primary health care with a specific program for health management. The inter-correlation between different risk factors shows a positive correlation. Our results conclude that both pre and post-heart patients should be more concerned about these risk factors. Moreover, this study can be used for further research and will increase people regarding their healthy choice.
